# Supplementary figures and images for: The Patent Ductus Arteriosus in Extremely Preterm Neonates Is More than a Hemodynamic Challenge: New Molecular Insights
Source: Biomolecules. 2022 Aug 25;12(9):1179. doi: 10.3390/biom12091179 (PMC9496182; doi:10.3390/biom12091179)

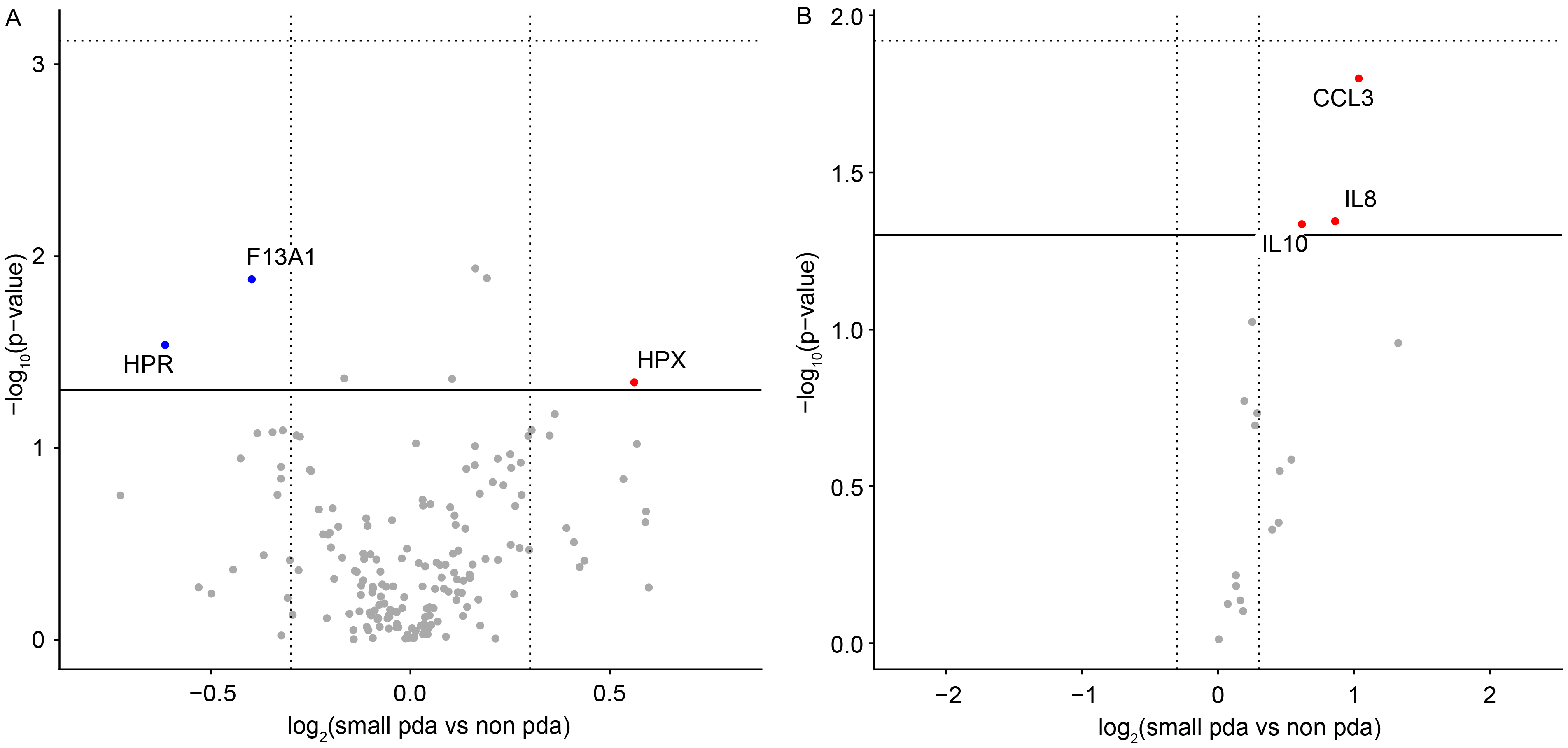

Supplement: Supplementary file 1 [file biomolecules-12-01179-s001.zip › Supplementary_figure_S1.png]

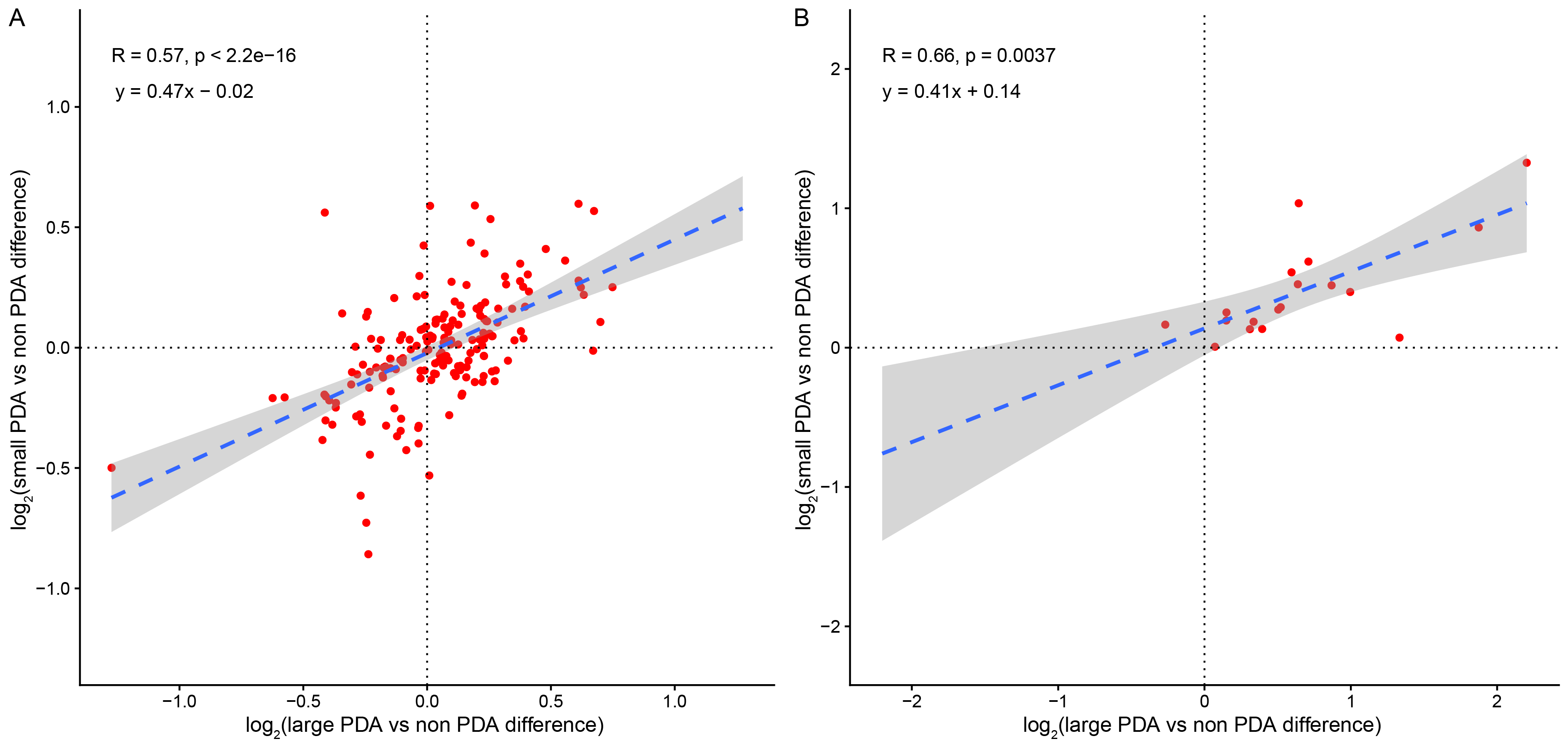

Supplement: Supplementary file 1 [file biomolecules-12-01179-s001.zip › Supplementary_figure_S2.png]

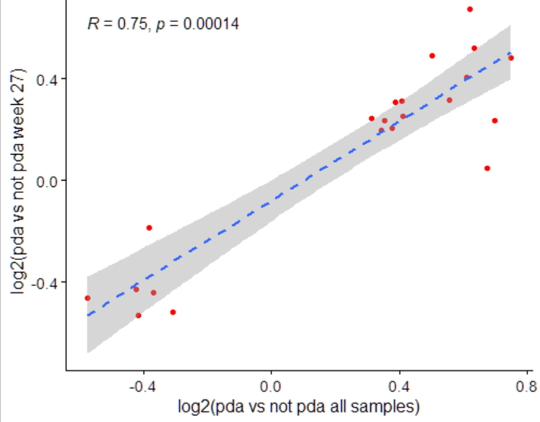

Supplement: Supplementary file 1 [file biomolecules-12-01179-s001.zip › Supplementary_figure_S3.png]

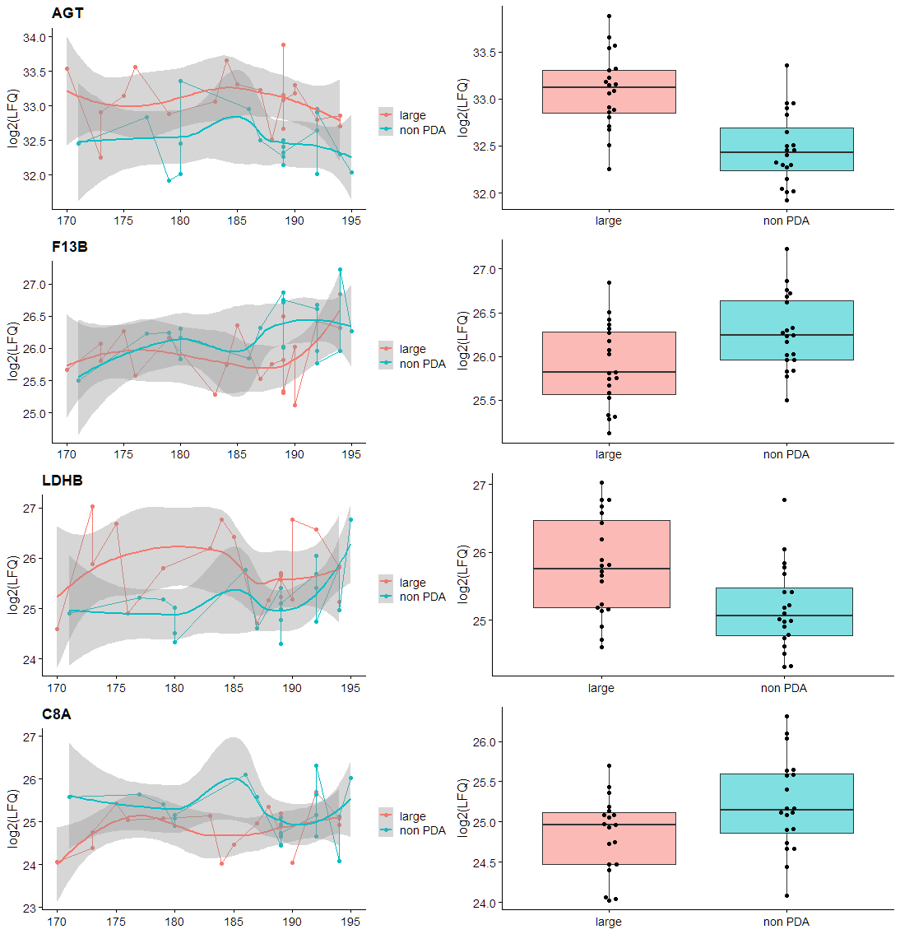

Supplement: Supplementary file 1 [file biomolecules-12-01179-s001.zip › Supplementary_figure_S4.png]

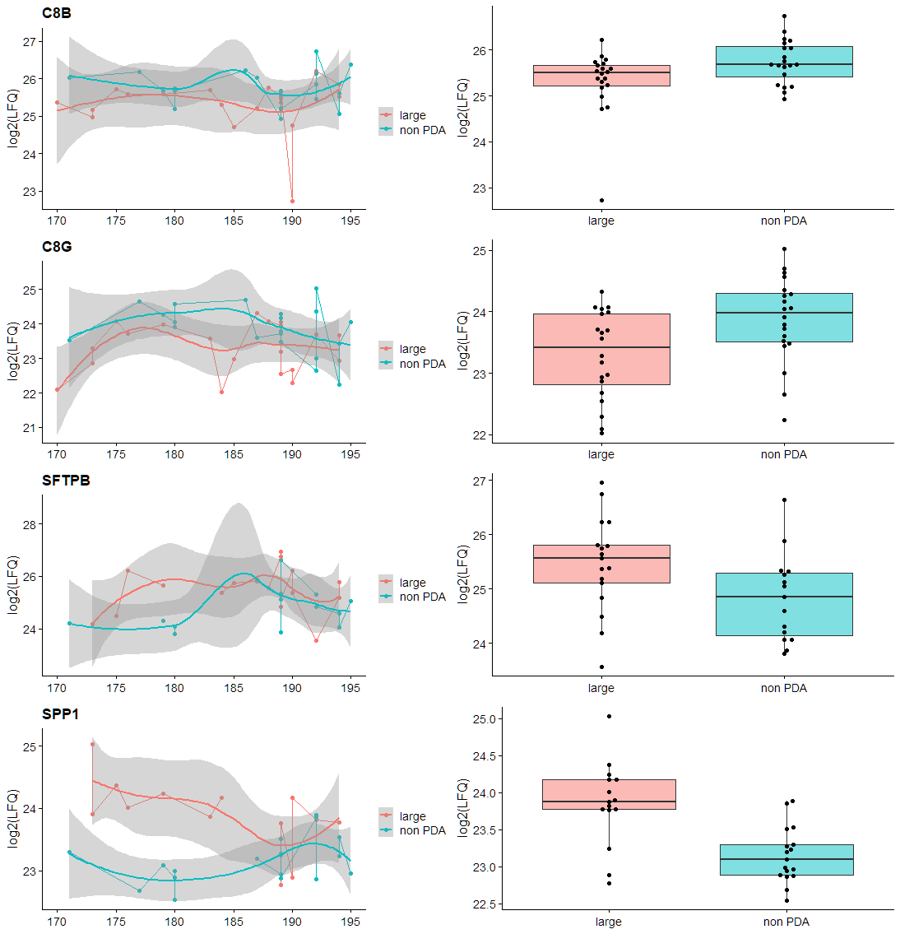

Supplement: Supplementary file 1 [file biomolecules-12-01179-s001.zip › Supplementary_figure_S5.png]

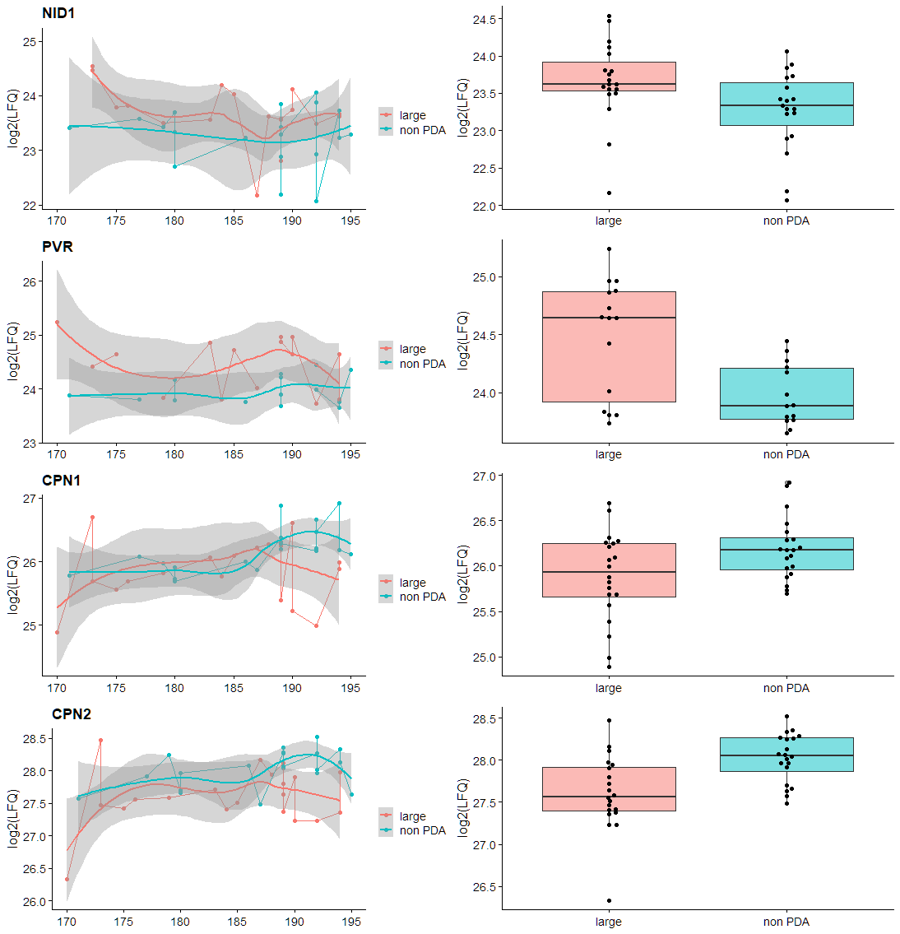

Supplement: Supplementary file 1 [file biomolecules-12-01179-s001.zip › Supplementary_figure_S6.png]

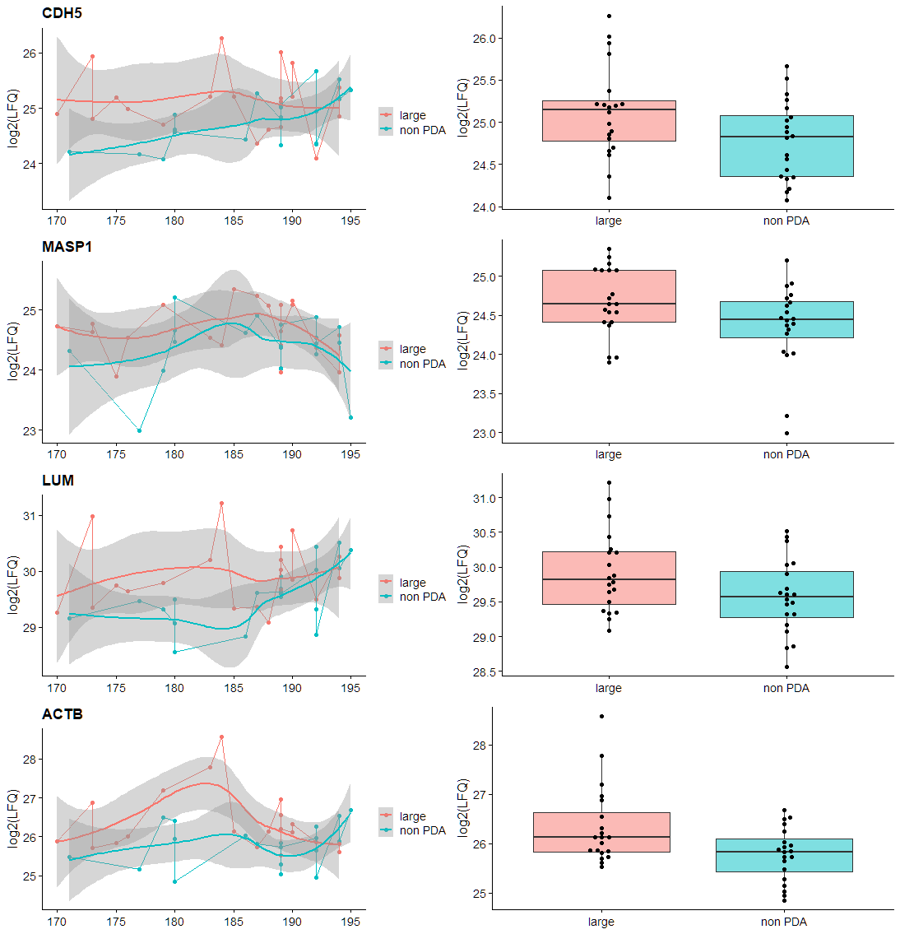

Supplement: Supplementary file 1 [file biomolecules-12-01179-s001.zip › Supplementary_figure_S7.png]

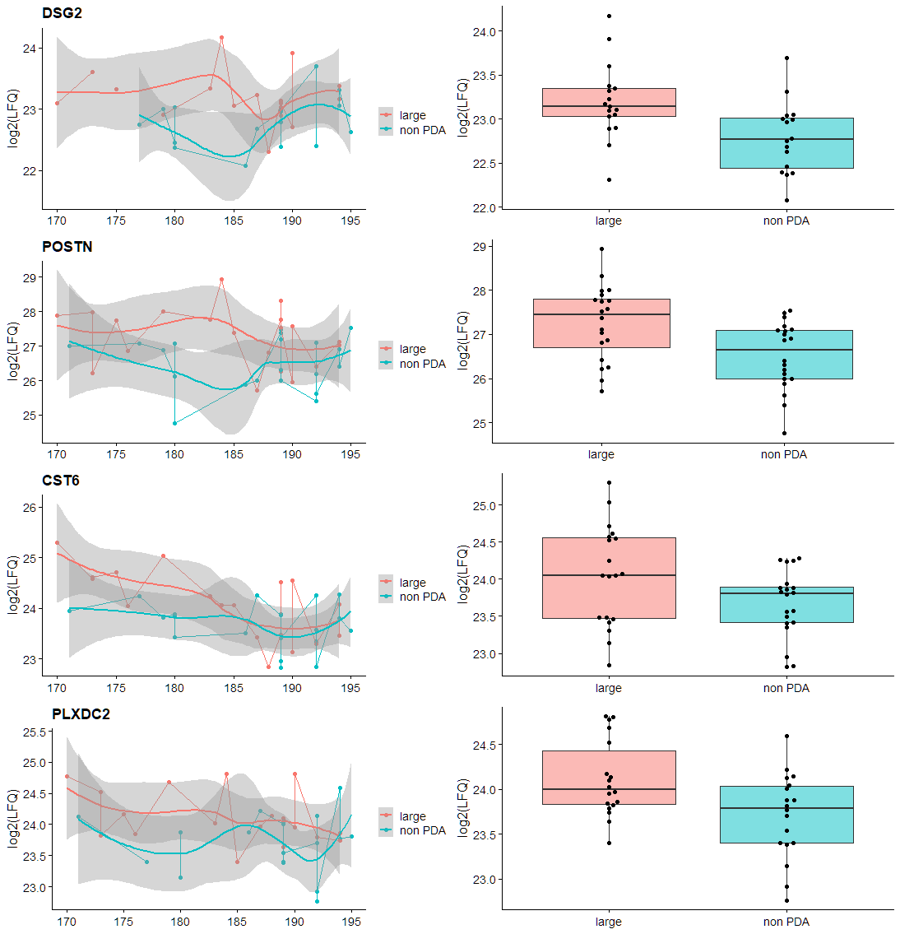

Supplement: Supplementary file 1 [file biomolecules-12-01179-s001.zip › Supplementary_figure_S8.png]

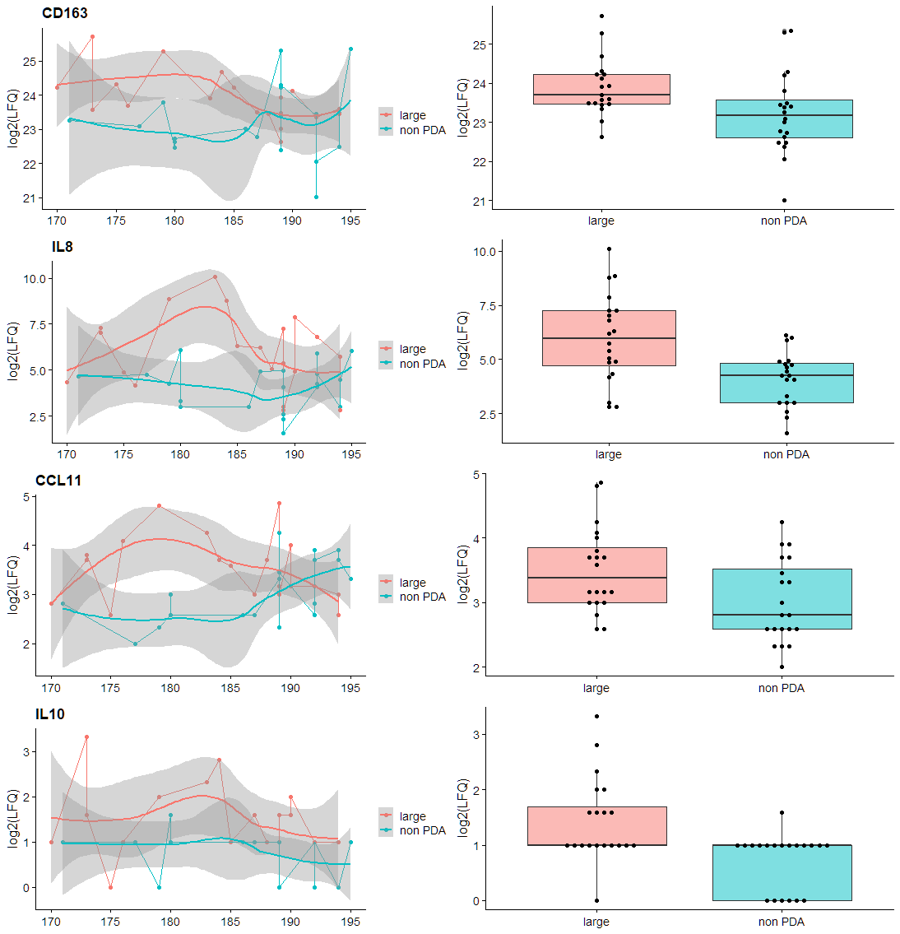

Supplement: Supplementary file 1 [file biomolecules-12-01179-s001.zip › Supplementary_figure_S9.png]

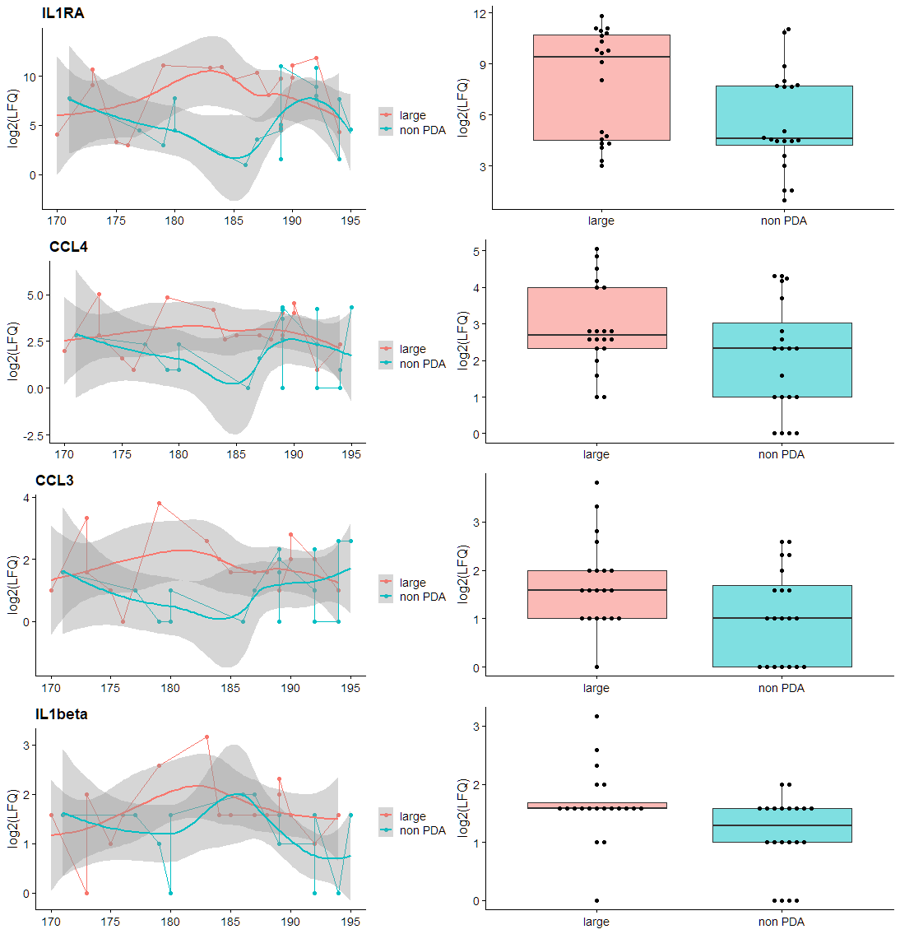

Supplement: Supplementary file 1 [file biomolecules-12-01179-s001.zip › Supplementary_figure_S10.png]

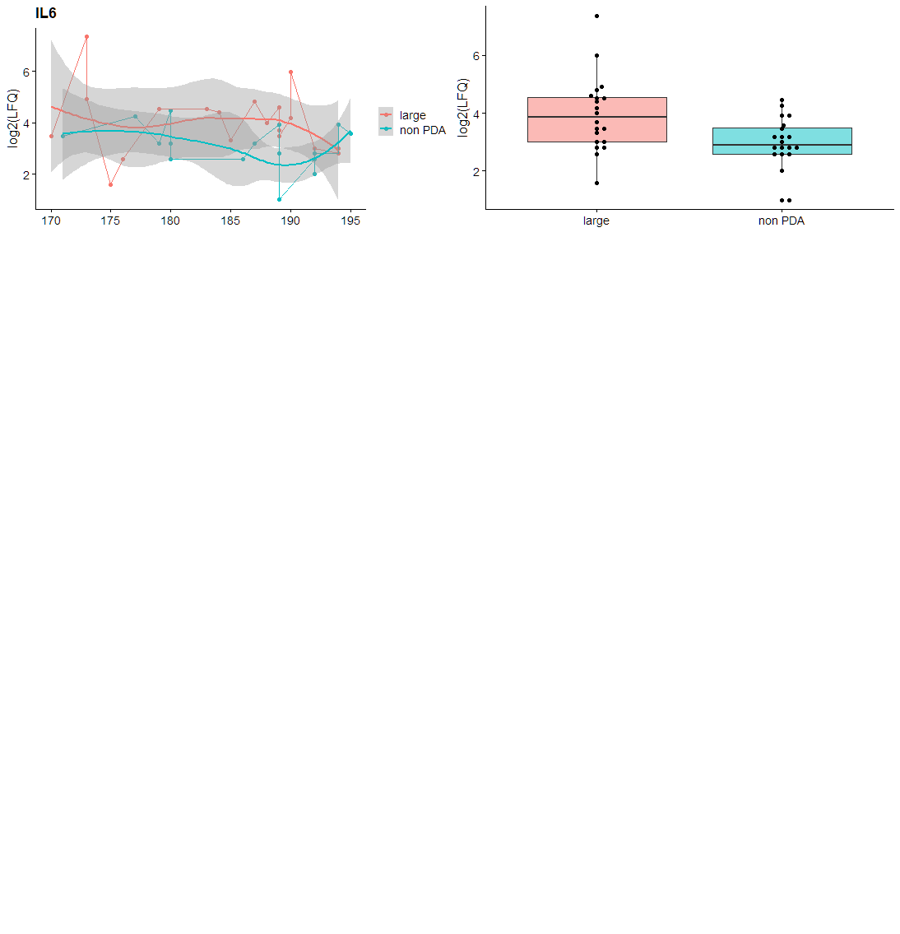

Supplement: Supplementary file 1 [file biomolecules-12-01179-s001.zip › Supplementary_figure_S11.png]

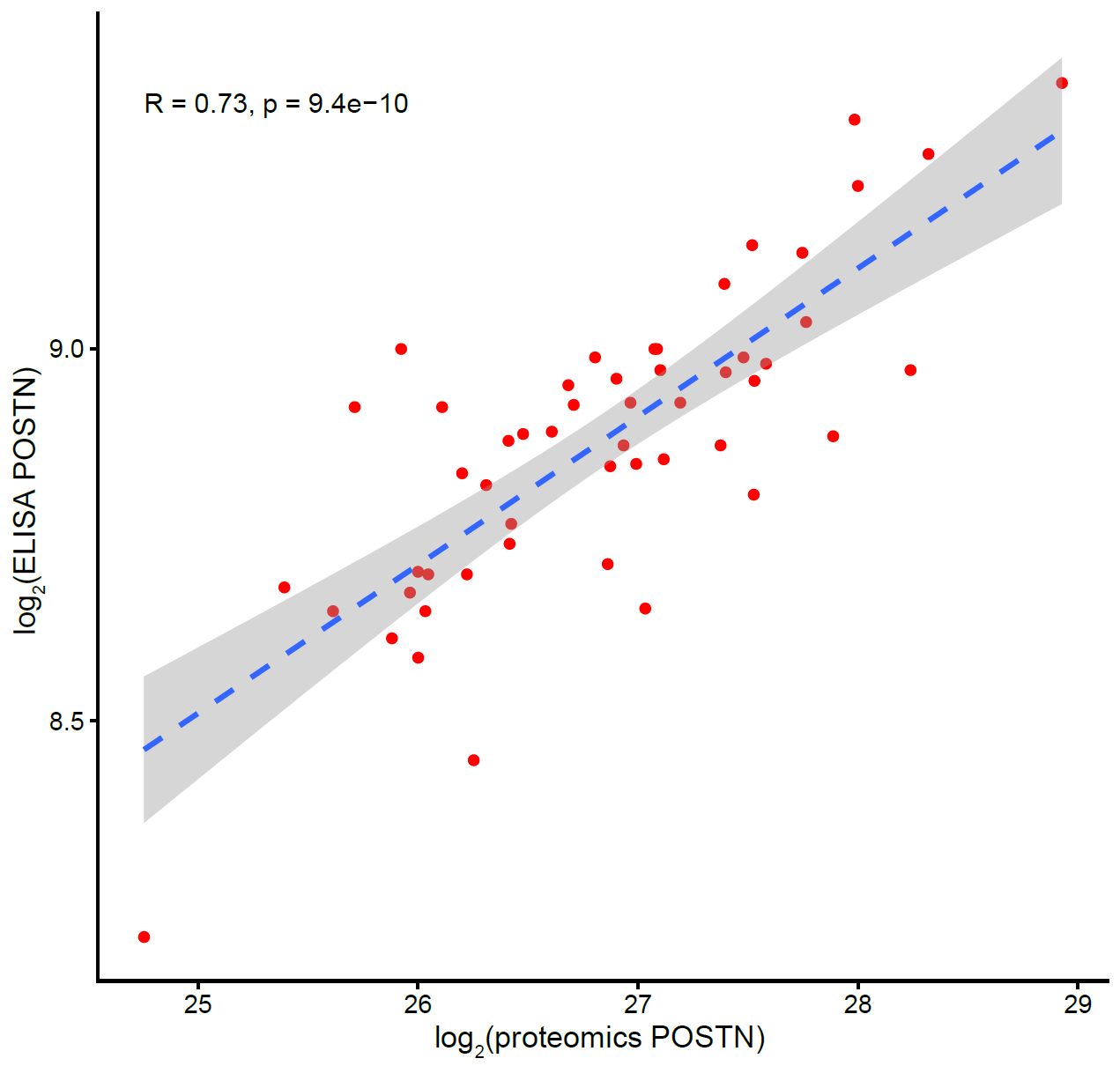

Supplement: Supplementary file 1 [file biomolecules-12-01179-s001.zip › Supplementary_figure_S12.png]
